# A Comparative Analysis of Graphene Versus Zirconia Fixed Dental Prostheses: An In Vitro Study

**DOI:** 10.7759/cureus.87360

**Published:** 2025-07-05

**Authors:** Shitij Srivastava, Abhidha Tripathi, Abhinav Shekhar, Anshuman Chaturvedi, Abhishek Singh, Shivansh M Saxena, Rabia Khan

**Affiliations:** 1 Department of Prosthodontics, Sardar Patel Post Graduate Institute of Dental and Medical Sciences, Lucknow, IND

**Keywords:** compressive strength, dental prosthesis, flexural strength, hardness test, prosthodontics

## Abstract

Objectives

This study set out to compare the mechanical behavior of graphene and zirconia in the context of their potential use in fixed dental prostheses. Specifically, we evaluated three critical properties: Rockwell hardness, to understand surface durability; compressive strength, to assess resistance to biting forces; and flexural strength, to examine performance under bending stress. To approximate real-world conditions, all samples were subjected to thermocycling, simulating the thermal changes typical of the oral cavity. The goal was to explore whether graphene could serve as a viable alternative to zirconia in restorative applications.

Methods

A systematic in vitro protocol was followed. Standardized specimens of graphene and zirconia were fabricated and subjected to 30,000 thermocycles between 5°C and 55°C. Mechanical tests were conducted using a universal testing machine. Rockwell hardness was measured using a standard durometer, while compressive and flexural strengths were evaluated through load-to-failure testing. Data were statistically analyzed using independent-sample t-tests, with significance defined at p<0.05.

Results

Zirconia showed markedly higher compressive strength compared to graphene, making it better suited for high-load areas of the mouth. Interestingly, the two materials performed similarly in Rockwell hardness, suggesting comparable surface durability. Flexural strength results were also close, with no significant difference, indicating that graphene may perform well under bending or tensile forces. These outcomes reaffirm zirconia's status as a robust material for prosthodontics while also opening the door for graphene as a lightweight, structurally capable alternative.

Conclusion

While zirconia continues to outperform in terms of compressive strength, graphene demonstrates meaningful potential due to its comparable flexural strength and hardness, along with the added advantage of being significantly lighter. These findings support further investigation into graphene's role in restorative dentistry, especially in cases where weight, design complexity, or aesthetics demand alternative materials. Future research should explore its biocompatibility, long-term performance, and integration with current dental systems.

## Introduction

Managing dental diseases using biomaterials is challenging because the materials must be placed within the oral cavity, where they are subjected to various environmental parameters such as moisture, temperature, pressure, food particles, and toothbrush abrasion. Dental materials may undergo mechanical failure or deterioration due to these harsh conditions, leading to treatment failure, inconvenience, and increased expense. The development of dental materials primarily focuses on their mechanical strength, biocompatibility, flexibility, ease of modification, and capacity to enhance the oral cavity's environmental conditions [1]. Improving patient outcomes in restorative dentistry depends heavily on the development and optimization of dental prosthesis materials. To restore oral function, replace lost or damaged teeth, and improve aesthetics, fixed dental prostheses such as crowns and bridges are necessary. Materials like ceramics and metals have been traditionally used for these purposes. However, new possibilities for advancing the discipline have emerged with the advent of innovative materials like graphene and zirconia.

Driven by the need to improve the lifetime, usability, and aesthetics of dental prostheses, the field of restorative dentistry has seen notable advances in materials science in recent years. Due to their remarkable mechanical qualities, graphene and zirconia have emerged as leading choices among the many materials assessed for their usefulness in permanent dental prostheses.

Graphene is a two-dimensional honeycomb lattice composed of a single layer of carbon atoms arranged in planar structures made up of hybridized sp² carbon atoms, each possessing distinct mechanical, electrical, chemical, and thermodynamic characteristics. One of the strongest and thinnest substances found in nature, graphene has a very large surface area (2600 m²/g) [2]. Its extraordinary qualities, such as great mechanical strength, flexibility, and electrical and thermal conductivity, have revolutionized several scientific fields since its discovery. Graphene is a promising material for dental applications due to its high tensile strength and stiffness, which might result in robust and long-lasting prosthetic solutions. The main forms being studied for both biomedical and non-biomedical uses are reduced graphene oxide and graphene oxide. However, due to their huge surface area, high surface energy, hydrophobicity, and low water dispersibility, the application possibilities of graphene sheets are still constrained [3].

Conversely, zirconia (zirconium dioxide) is a well-known substance in dental prostheses. Zirconia has grown in popularity as a material for permanent dental prostheses due to its high compressive strength, biocompatibility, and cosmetic attributes [4]. By adjusting the crystalline structure of zirconia, its properties may be further improved; distinct phases of the material have varying mechanical characteristics. Comparing zirconia to newer materials like graphene is relevant due to its adaptability and track record of success.

It is crucial to assess and compare the compressive strength, flexural strength, and Rockwell hardness of graphene and zirconia to determine which material is best suited for dental prostheses. These properties are essential markers of a material's efficacy in a therapeutic context.

Rockwell hardness measures a material's resistance to indentation. When evaluating the durability and wear resistance of dental prosthetic materials, it is an important consideration. A material's capacity to endure mechanical stress and resist deformation, reflected in its higher Rockwell hardness, is critical to preserving the integrity of dental restorations under functional loads.

Compressive strength refers to the maximum load a material can withstand while being compressed. In the context of dental prostheses, high compressive strength is vital for withstanding the occlusal forces exerted during normal chewing and biting activities. Materials with low compressive strength may fail or deform under such stresses, leading to premature prosthesis failure.

Flexural strength measures a material's ability to resist deformation under load. It is particularly relevant for fixed dental prostheses, which are subjected to bending forces during functional use. Adequate flexural strength ensures that the prosthesis can endure the mechanical stresses encountered in the oral cavity without fracturing or deforming.

## Materials and methods

Ethical approval

The present in vitro study was conducted across three main sites following the ethical standards outlined in the Declaration of Helsinki for research involving materials related to humans: (1) sample preparation and standardization at Panna Lab, Lucknow; (2) mechanical property testing of the samples at the Central Institute of Petrochemicals and Technology, Lucknow; and (3) final analysis and interpretation of the results at the Sardar Patel Post Graduate Institute of Dental and Medical Sciences, Lucknow. Although no human participants or animal subjects were involved, ethical clearance was obtained to ensure compliance with institutional research policies.

The study protocol was reviewed and approved by the Institutional Ethical Committee of Sardar Patel Post Graduate Institute of Dental and Medical Sciences (approval number: PROSTHO/01/222331/IEC), dated 27/04/2023. All procedures involving the use of dental materials were performed following approved laboratory safety and research guidelines.

Study design and sample division

This in vitro experimental study was designed to evaluate and compare the mechanical properties of graphene and zirconia, two materials commonly used in the fabrication of fixed dental prostheses. A previous study reported a mean density of 2.2 g/cm³ for graphene and 5.7 g/cm³ for zirconia [5]. Drawing from these findings, the sample size was calculated using the formula proposed by Charan and Biswas for comparing two independent means [6]: $n = \left( \frac{r + 1}{r} \right) \cdot \left( \frac{SD^2 \cdot (Z_{\beta} + Z_{\alpha/2})^2}{d^2} \right)$.

**Figure 1:**
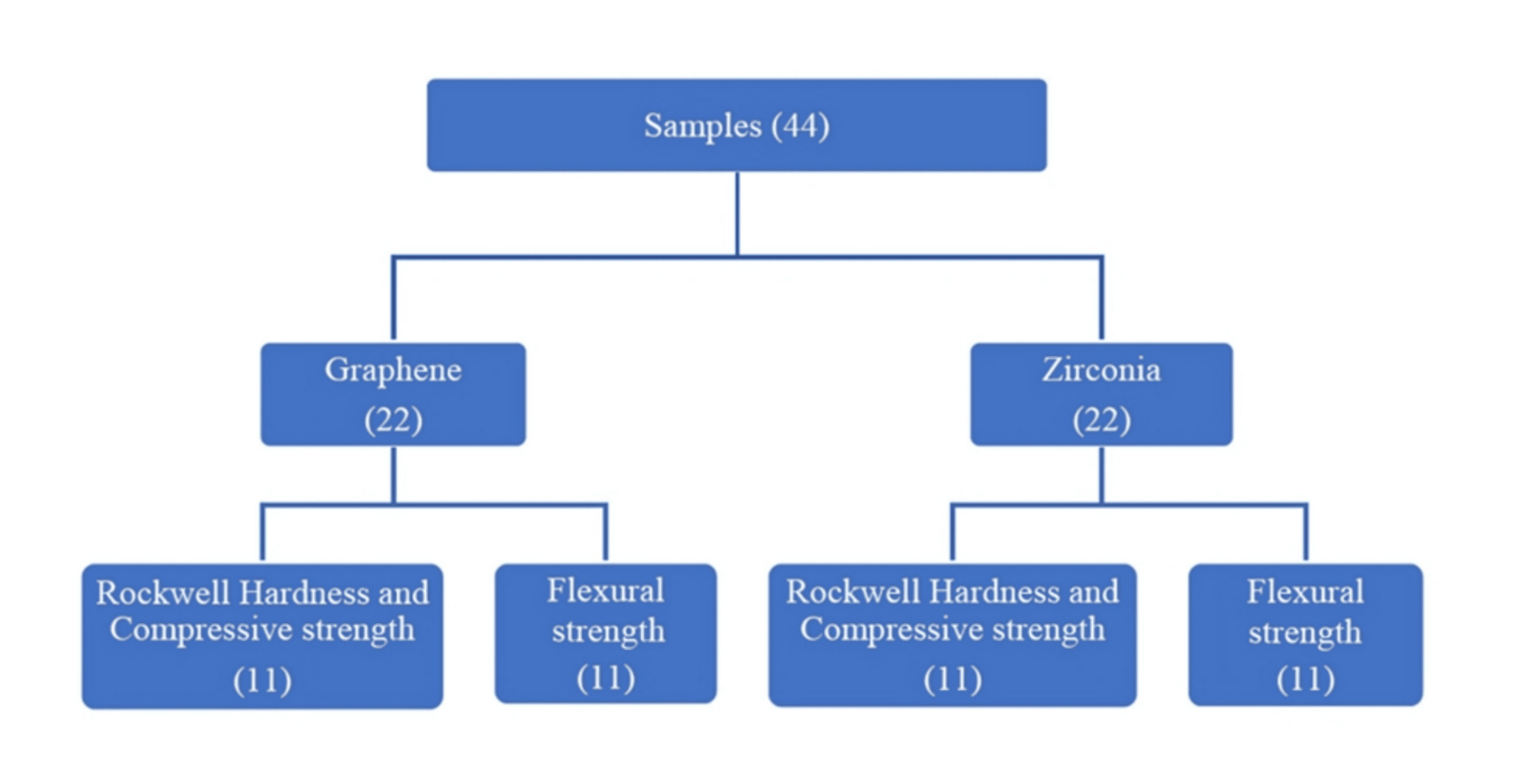
Distribution of sample groups (n=44) for the mechanical property evaluation of graphene and zirconia materials

Forty-four samples were found to be adequate to detect a statistically significant difference between the two groups with a confidence level of 95% (α=0.05) and 80% power, assuming an effect size of 1.0 (Figure 1).

This study design ensured balanced comparison and statistical validity for evaluating the selected mechanical properties across the two materials (Figure 1).

Methodology

The methodology of this study was divided into five distinct phases to systematically evaluate the mechanical properties of graphene and zirconia.

Phase I: Graphene Specimen Preparation

Graphene specimens were prepared from commercially available graphene nano-reinforced biopolymer discs (G-CAM), designed specifically for permanent dental restorations with natural aesthetics. These discs, compatible with dental milling machines (95 mm or 98.5 mm diameter), were sectioned into cuboidal samples measuring 20×10×5 mm, as recommended by the manufacturer. This set of specimens was categorized as group A for subsequent testing.

Phase II: Zirconia Specimen Preparation

Zirconia specimens were fabricated according to the manufacturer's guidelines, involving an initial plastic shaping phase followed by sintering to achieve full densification. The sintered zirconia was then shaped into identical cuboidal samples matching the dimensions of the graphene specimens (20×10×5 mm). To standardize the surface finish, all zirconia samples were polished using 1200-grit abrasive paper. These specimens were designated as group B.

Phase III: Standardization

All samples underwent careful dimension verification using precision instruments such as calipers and micrometers to ensure compliance with the required size specifications. Baseline mechanical properties, including Rockwell hardness, compressive strength, and flexural strength, were recorded prior to any aging processes, providing initial reference data for comparison.

Phase IV: Aging of Samples

To simulate the thermal stresses materials experience in the oral environment, all specimens were subjected to thermocycling according to ISO/TS 11405 standards. This involved alternately immersing samples in distilled water baths at 5°C and 55°C, with each temperature held for 13 seconds, completing a total of 30,000 cycles. After thermocycling, the samples were returned to room temperature in preparation for mechanical testing.

Phase V: Mechanical Testing Procedures

Mechanical characterization consisted of Rockwell hardness, compressive strength, and flexural strength testing.

Rockwell hardness testing was performed with a diamond cone indenter applying a minor preload followed by a major load consistent with the Rockwell C scale. Indentation depth measurements were used to calculate hardness values. The Rockwell hardness number was derived from the difference in indentation depths between the minor and major loads as follows: $\text{Rockwell Hardness Number} = 100 - \left( \frac{\text{Depth Difference}}{0.002 \, \text{mm}} \right)$. Here, the depth difference is expressed in multiples of 0.002 mm.

Compressive strength was tested by gradually applying a load at 0.5 mm/min until specimen failure. The maximum load at failure was recorded, and compressive strength was calculated using the equation $\text{Compressive Strength} = \frac{\text{Maximum Load}}{\text{Cross-Sectional Area}}$ where the cross-sectional area=20×10 mm=200 mm^2^.

For flexural strength, a four-point bending test was conducted at a crosshead speed of 1 mm/min until fracture. The flexural strength was calculated using the equation $\text{Flexural Strength} = \frac{3FL}{2bd^2}$ where $F$ is the maximum load, $L$ is the span length between supports, $b$ is the specimen width, and $d$ is the specimen depth (thickness).

Each mechanical test was performed on 11 specimens per material to ensure the accuracy and reproducibility of results. All mechanical tests were conducted under controlled laboratory conditions at room temperature (23±1°C) and relative humidity of 50± 5%, ensuring consistency during testing procedures.

Data collection and analysis

The data collection procedure for this study involved a systematic and detailed approach to ensure accuracy and consistency in evaluating the two materials used in the fabrication of fixed dental prostheses, that is, graphene and zirconia, which were assessed for their mechanical properties. These properties were evaluated by subjecting the samples to a universal testing machine and measuring Rockwell hardness, compressive strength, and flexural strength, which are considered the most crucial mechanical characteristics of dental materials.

To minimize potential bias during the evaluation phase, single blinding was implemented, whereby the assessor responsible for measuring the mechanical outcomes was blinded to the material group allocation. This ensured that the measurement and interpretation of results remained objective and unaffected by preconceived expectations.

Quantitative data were expressed as mean±standard deviation (SD) for continuous variables, providing a clear understanding of the central tendency and variability within each group. Additionally, percentages and frequencies were used to summarize the distribution of samples across material groups, ensuring transparency in sample allocation.

All statistical analyses were conducted using IBM SPSS Statistics for Windows, Version 21.0 (IBM Corp., Armonk, New York, United States). Descriptive statistics were calculated to establish baseline trends for each mechanical parameter across the graphene and zirconia groups.

To test for differences in mean values between the two groups, Student's t-test was applied when data were normally distributed, ensuring robust comparisons between independent sample sets. For datasets that did not meet the assumptions of normality, appropriate non-parametric alternatives were considered to maintain statistical validity.

In addition to comparing group means, bivariate correlation analysis was performed using the Pearson product-moment correlation coefficient. This was used to assess the strength and direction of the linear relationship between different mechanical properties across samples. Interpretation of correlation strength was based on standard thresholds, ranging from weak to very strong correlations.

To determine the relevance of the observed differences, a p-value of <0.05 was considered statistically significant. More stringent thresholds (e.g., p<0.01 and p<0.001) were used to denote high and very high levels of significance, respectively. This allowed the findings to be interpreted with confidence regarding their potential clinical and material implications.

## Results

In the present study, 22 (50%) specimens were prepared using graphene material; these specimens were categorized as group A. The remaining 22 (50%) specimens were prepared using zirconia material; these specimens were categorized as group B (Table 1).

**Table 1:** Group-wise distribution of the specimens (n=44)

S. no.	Group	Material	Specimen (n)	Percentage (%)
1	Group A	Graphene	22	50
2	Group B	Zirconia	22	50
	Total		44	100

The macro-hardness of group A specimens ranged from 62.14 to 68.67 Newton, while that of group B specimens was from 44.44 to 47.38 Newton. The mean macro-hardness of group A was found to be significantly higher than that of group B (64.74±1.68 vs. 45.42±0.67 Newton) (Table 2).

**Table 2:** Intergroup comparison of Rockwell hardness (in Newton) This table presents the minimum, maximum, mean, and standard deviation (SD) of Rockwell hardness values for group A and group B specimens (n=22 each). Group A demonstrated significantly higher macro-hardness compared to group B. Statistical analysis using Student's t-test revealed a highly significant difference between the groups (t=50.146; p<0.001).

Group	Specimen (n)	Minimum	Maximum	Mean	SD
Group A	22	62.14	68.67	64.74	1.68
Group B	22	44.44	47.38	45.42	0.67
Total	44	44.44	68.67	55.07	9.86

Compressive strength of group A specimens ranged between 72.98 and 76.38 Newton, and its mean value was found to be 74.85±0.96. On the other hand, the range of compressive strength of group B specimens was between 1299 and 1510 Newton, and its mean value was 1407.18±56.41 Newton. The difference in mean compressive strength of group A and group B specimens was found to be statistically significant (Table 3).

**Table 3:** Intergroup comparison of compressive strength (in Newton) This table summarizes the minimum, maximum, mean, and standard deviation (SD) of compressive strength values for group A and group B (n=22 each). Group B exhibited significantly higher compressive strength compared to group A. Statistical analysis using Student's t-test showed a highly significant difference between the groups (t=-110.771; p<0.001).

Group	Specimen (n)	Minimum	Maximum	Mean	SD
Group A	22	72.98	76.38	74.85	0.96
Group B	22	1299	1510	1407.18	56.41
Total	44	72.98	1510	741.02	675.02

Flexural strength of group A specimens ranged between 1245 and 1654 MPa, and its mean value was 1446.64±93.95 MPa, while flexural strength of group B specimens ranged between 934 and 1764 MPa, and its mean value was 1182.64±243.47 MPa. The flexural strength of group A specimens was found to be significantly higher as compared to that of group B (Table 4).

**Table 4:** Intergroup comparison of flexural strength (in MPa) This table presents the minimum, maximum, mean, and standard deviation (SD) of flexural strength values for group A and group B specimens (n=22 each). Group A demonstrated significantly higher flexural strength than group B. Statistical analysis using Student's t-test revealed a significant difference between the groups (t=4.745; p<0.001).

Group	Specimen (n)	Minimum	Maximum	Mean	SD
Group A	22	1245	1654	1446.64	93.95
Group B	22	934	1764	1182.64	243.47
Total	44	934	1764	1314.64	226.03

**Figure 2:**
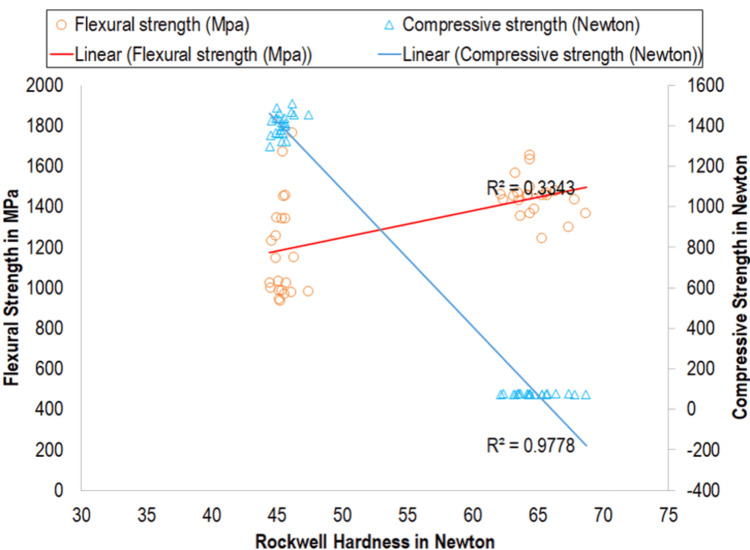
Correlation between Rockwell hardness (N), flexural strength (MPa), and compressive strength (N) Scatter plot showing the relationship between Rockwell hardness (in Newton) and two mechanical properties: flexural strength (in MPa, left Y-axis) and compressive strength (in Newton, right Y-axis). Flexural strength is represented by orange circles with a fitted linear trendline (red), while compressive strength is shown using blue triangles with a corresponding linear fit (blue). The coefficient of determination (R²) indicates a moderate positive correlation (R²=0.3343) between Rockwell hardness and flexural strength and a strong negative correlation (R²=0.9778) between Rockwell hardness and compressive strength.

Among overall specimens, correlations of Rockwell hardness with compressive strength and flexural strength were found to be statistically significant. The level of correlation between Rockwell hardness and compressive strength was found to be very strong, while the correlation between Rockwell hardness and flexural strength was of a moderate level (Table 5; Figure 2).

**Table 5:** Correlation of Rockwell hardness, compressive strength, and flexural strength This table shows Pearson's correlation coefficients (r) between Rockwell hardness (N) and compressive strength (N) and flexural strength (MPa). A very strong negative correlation was observed between hardness and compressive strength (r=-0.989; p<0.001), while a moderate positive correlation was found with flexural strength (r=0.578; p<0.001). Both correlations were statistically highly significant.

S. no.	Physical characteristics	Rockwell hardness			
		Pearson's correlation		Statistical significance	
		"r"	Level of correlation	"p"	Level of significance
1	Compressive strength (N)	-0.989	Very strong	<0.001	Highly significant
2	Flexural strength (MPa)	0.578	Moderate	<0.001	Highly significant

For group A specimens, the correlation of Rockwell hardness with compressive strength was found to be weak and statistically non-significant, while the correlation of Rockwell hardness and flexural strength was of a mild level but statistically non-significant (Table 6).

**Table 6:** Correlation of Rockwell hardness, compressive strength, and flexural strength of group A specimens This table presents Pearson's correlation between Rockwell hardness and compressive strength (N) and flexural strength (MPa) for group A specimens. No significant correlation was observed with compressive strength (r=-0.001; p=0.997) or flexural strength (r=-0.308; p=0.164), indicating weak and statistically non-significant relationships.

S. no.	Physical characteristics	Rockwell hardness			
		Pearson's correlation		Statistical significance	
		"r"	Level of correlation	"p"	Level of significance
1	Compressive strength (N)	-0.001	No or weak	0.997	Non-significant
2	Flexural strength (MPa)	-0.308	Mild	0.164	Non-significant

For group B specimens, a mild and statistically significant correlation between Rockwell hardness and compressive strength was found. The correlation between Rockwell hardness and flexural strength was found to be weak and statistically non-significant (Table 7).

**Table 7:** Correlation of Rockwell hardness, compressive strength, and flexural strength of group B specimens Correlation analysis between Rockwell hardness and mechanical properties (compressive strength and flexural strength) of group B specimens. Pearson's correlation coefficient (r) indicates the strength and direction of the correlation, while the p-value (p) denotes the statistical significance. A significant positive mild correlation was observed between hardness and compressive strength (r=0.437; p=0.042). No significant correlation was found between hardness and flexural strength (r=0.084; p=0.709).

S. no.	Physical characteristics	Rockwell hardness			
		Pearson's correlation		Statistical significance	
		"r"	Level of correlation	"p"	Level of significance
1	Compressive strength (N)	0.437	Mild	0.042	Significant
2	Flexural strength (MPa)	0.084	No or weak	0.709	Non-significant

## Discussion

This study sought to investigate the difference in the mechanical properties of two of the most novel materials used in fixed dental prostheses, i.e., graphene and zirconia, specifically evaluating the Rockwell hardness, compressive strength, and flexural strength of the materials. The effects of thermocycling on the mechanical properties of these materials are of significant importance in determining their longevity and performance in an oral environment. The central hypothesis posited the application of graphene and zirconia in day-to-day dentistry and their success in clinical applications, including their mechanical strength, biocompatibility, and aesthetic properties. By comparing these mechanical properties, this study aimed to contribute valuable insights into optimizing the best material possible for restorative dentistry and its long-term success in an oral environment.

Two groups of samples were fabricated after following standardization procedures. In group A, graphene cuboidal samples were fabricated. In group B, zirconia cuboidal samples were fabricated.

Testing of the samples and intergroup comparisons

The samples from groups A and B were subjected to mechanical testing under a universal testing machine, and the tests for Rockwell hardness, compressive strength, and flexural strength were performed. The results of the ANOVA test revealed that among overalls, the correlation of Rockwell hardness with compressive strength and flexural strength was found to be statistically significant. The level of correlation between Rockwell hardness and compressive strength was found to be very strong, while the correlation between Rockwell hardness and flexural strength was of a moderate level.

For group A specimens, the correlation of Rockwell hardness with compressive strength was found to be weak and statistically non-significant, while the correlation of Rockwell hardness and flexural strength was of a mild level but statistically non-significant. For group B specimens, a mild and statistically significant correlation between Rockwell hardness and compressive strength was found. The correlation between Rockwell hardness and flexural strength was found to be weak and statistically non-significant.

These results support the hypothesis that while graphene outperforms zirconia in Rockwell hardness, the flexural strength and compressive strength of the zirconia are significantly higher. These findings align with previous studies, which stated that the difference in the amount of stress, deflection, strain, and deformation on using different materials and configurations in the pontic and connector area of the dental bridge was evaluated [5]. The study highlighted higher values of normal stress, deflection, equivalent elastic strain, and total deformation in graphene-based bridges than in the zirconia-based bridges. Additionally, a study concluded that the increase in flexural strength of all ceramics incorporated with graphene nanoparticles supports research work by demonstrating that all ceramics incorporated with graphene nanoparticles showed superior flexural strength [7].

A thorough investigation highlighted graphene-based materials as a promising candidate for dentistry materials, which have been widely used in dentistry research owing to their cell differentiation and antibacterial properties [8]. Their review summarized recent advances in expanding the types of graphene-based materials and the studies about dentistry-related properties, deepening the understanding of categories of graphene-based materials. With the development of the application of graphene in dentistry, there are still some challenges remaining to be tackled until the final commercialization. Graphene and its derivatives will be of great interest for a long time in the dental field. Although there are some limitations in the real clinical usage of dentistry, graphene, as a more reliable and friendly biomaterial, can prompt more effective dental treatments in the near future [9].

The utilization of graphene in reconstructive dentistry has been a significant advancement. Due to its 2D structure and exceptional characteristics, graphene has demonstrated its potential in prosthodontics and implant dentistry [10]. Incorporation of graphene-based nanomaterials can enhance the chemical, physical, and mechanical properties of biomaterials, thus offering promising options for innovative therapeutic approaches in dentistry [11].

Compressive strength is a crucial indicator of success because a high compressive strength is necessary to resist masticatory and parafunctional forces [12]. According to Phillips, compressive strength is the capacity of a material or structure to withstand axially directed pushing forces. It provides data of force versus deformation for the conditions of the test method [13].

Hardness is a measure of how well materials resist localized deformation. Hardness is a consolidated manifestation of different properties like compressive strength, proportional limit, and ductility [14]. Test methods for hardness are on the basis of the ability of a material surface to resist penetration by a specified indenter. Rockwell hardness is a rapid testing method and is used as a quality control measure. It uses a diamond cone or steel ball and applies a minor load of 3 kgf; subsequently, a major load exceeding 10 kgf is applied. For experiments with dental materials, 30 kgf is the preferred final load. Based on the changes in depth that have occurred between the minor and major loads, the Rockwell hardness number is calculated.

In addition to the abovementioned tests, our study has conducted thermocycling on the dental materials to simulate clinical service. Thermal cycling is one of the most widely used procedures to simulate the physiological aging experienced by biomaterials in clinical practice [15]. The long-term success of modern dental restoratives is limited by their durability in the oral environment [16]. Longevity and efficiency are characteristics that should ideally be provided by each product; however, these properties are still goals to be achieved. A study on the mechanical properties of a novel graphene-reinforced polymethyl methacrylate (PMMA)-based dental restorative material revealed that graphene-reinforced PMMA-based dental restorative materials have a low compressive strength and high flexural strength, which is similar to the results of the study [17].

The study concluded that graphene demonstrated superior performance in Rockwell hardness compared to zirconia. However, zirconia exhibited significantly higher values in both flexural strength and compressive strength when compared to graphene. These findings suggest that while graphene excels in hardness, zirconia has a marked advantage in its ability to withstand bending and compressive forces. Such results underscore the distinct material properties of these substances and highlight the need for careful selection based on specific application requirements.

Clinical implications

This study provides valuable insights into the performance of zirconia- and graphene-based dental materials under thermocycling conditions that simulate the oral environment. Graphene, as a nano-reinforced biopolymer, demonstrated favorable mechanical and aesthetic properties, suggesting its potential for anterior restorations. However, its sensitivity to thermal stress warrants clinical caution in long-term applications. Zirconia maintained its high strength and durability post-thermocycling, though surface alterations emphasize the need for optimal fabrication and surface treatment. These findings underscore the importance of accelerated aging protocols in predicting material longevity and guiding evidence-based material selection in restorative dentistry.

Limitations of the study

This in vitro study, while methodologically robust, presents several limitations. Laboratory conditions cannot fully replicate the complexity of the oral environment, where factors such as saliva composition, enzymatic activity, microbial presence, and dynamic masticatory forces may influence material performance. Although the thermocycling protocol followed ISO/TS 11405:39 guidelines, it may not precisely reflect the frequency or extremes of intraoral temperature fluctuations encountered clinically.

Standardized specimen dimensions (20×10×5 mm) were used for mechanical testing, which may not accurately represent clinical restoration geometries or stress distributions at bonding interfaces. The investigation was limited to two materials, namely, graphene-reinforced biopolymer and zirconia, reducing generalizability across other dental materials with varying compositions and manufacturing protocols.

Additionally, the study focused on select mechanical properties (Rockwell hardness, compressive strength, and flexural strength), omitting other clinically relevant attributes such as wear resistance, fracture toughness, and biocompatibility. Finally, while thermocycling simulates accelerated aging, it does not capture the full scope of long-term degradation processes observed in vivo. Future studies incorporating clinical evaluations and broader material comparisons are recommended to enhance the translational value of these findings.

## Conclusions

This study systematically evaluated the mechanical properties of graphene and zirconia through a structured five-phase methodology, including specimen preparation, dimensional verification, thermocycling, and mechanical testing of Rockwell hardness, compressive strength, and flexural strength using a universal testing machine. The findings highlight distinct material-specific responses to thermocycling, emphasizing the critical role of thermal stability in dental restorative applications.

Graphene specimens (group A), characterized by their biopolymer-reinforced composition and superior aesthetics, demonstrated significant adaptability and potential for permanent dental restorations. Zirconia specimens (group B), produced via sintering, showed strong mechanical properties but exhibited performance variations after thermocycling, aligning with prior literature.

The thermocycling protocol, based on ISO/TS 11405:2015 standards, effectively simulated oral thermal fluctuations and revealed key insights into material durability. Results were consistent with previous studies demonstrating that thermal aging can alter surface microstructure and degrade mechanical strength. Mechanical testing further quantified these effects, providing comparative data that inform material selection in clinical dentistry.

Overall, this work underscores the influence of intrinsic material composition and extrinsic aging factors on restorative performance. The innovative properties of graphene and the proven resilience of zirconia each offer distinct advantages and limitations, guiding future biomaterial development and clinical applications.
